# Supplementary material for: Using nutation-frequency-selective pulses to reduce radio-frequency field inhomogeneity in solid-state NMR
Source: Magn Reson (Gott). 2020 Sep 9;1(2):187–95. doi: 10.5194/mr-1-187-2020 (PMC10500731; doi:10.5194/mr-1-187-2020)
Supplement: The supplement related to this article is available online at: https://doi.org/10.5194/mr-1-187-2020-supplement. [file mr-1-187-supplement.pdf]

Supplement of Magn. Reson., 1, 187–195, 2020
https://doi.org/10.5194/mr-1-187-2020-supplement
© Author(s) 2020. This work is distributed under
the Creative Commons Attribution 4.0 License.

Open Access
MAGNETIC RESONANCE

Supplement of

Using nutation-frequency-selective pulses to reduce radio-frequency field inhomogeneity in solid-state NMR

Kathrin Aebischer et al.

Correspondence to: Matthias Ernst (maer@ethz.ch)

The copyright of individual parts of the supplement might differ from the CC BY 4.0 License.

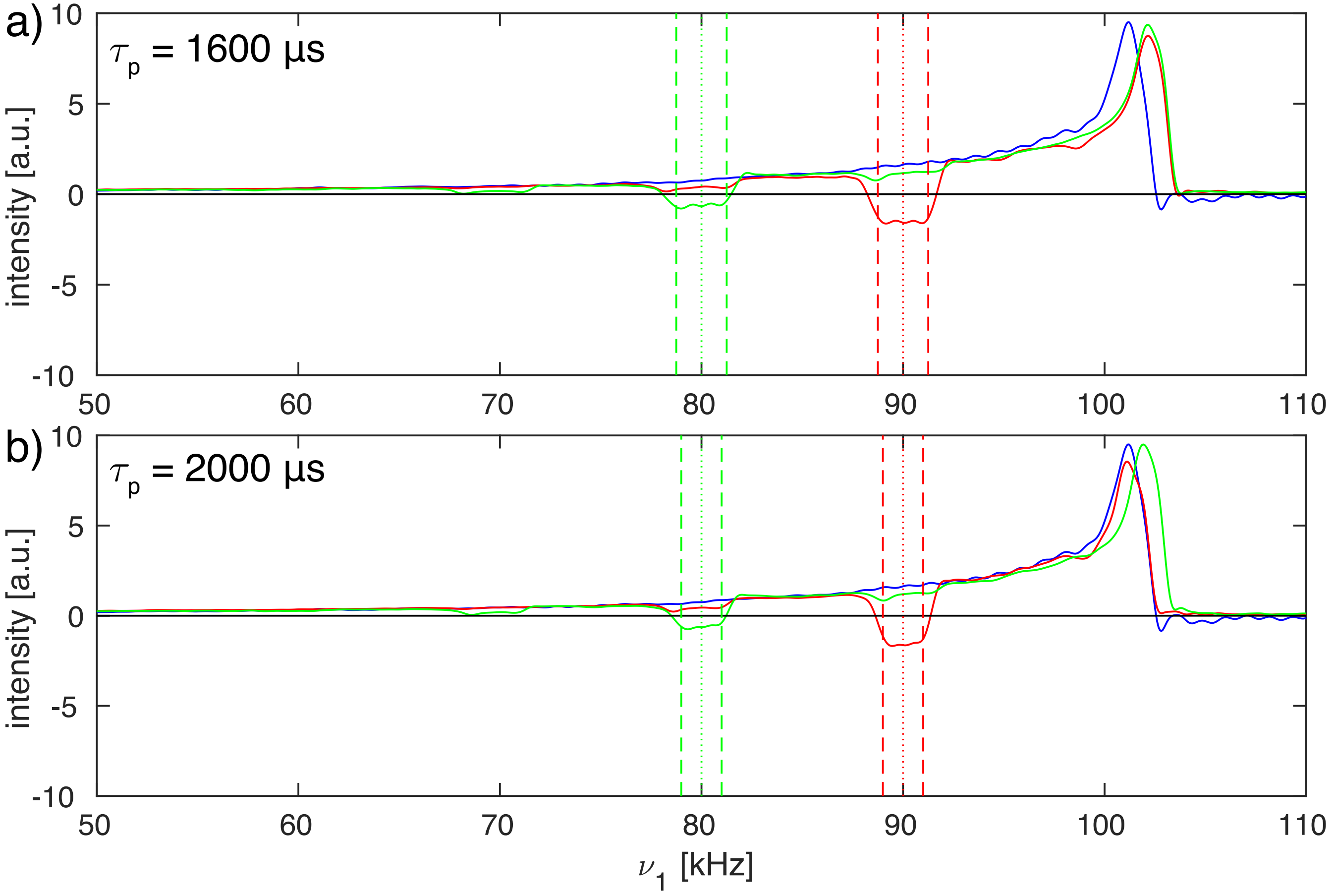

Figure S01: Proton nutation spectra of adamantane spinning at 10 kHz at 600 MHz proton resonance frequency using a Bruker 2.5 mm MAS probe. In blue, a standard nutation experiment is shown with a nominal rf-field amplitude of 100 kHz determined by the zero-crossing of a 5 µs π pulse. The nutation spectra in green and red were preceded by an I-BURP-2 pulse of length a) 1600 µs, b) 2000 µs using a modulation frequency of 80 kHz (green) and 90 kHz (red), respectively. In contrast to Fig. 4 in the main paper, there is only a very small shift in the theoretical and experimental inversion ranges. However, one can clearly see that there are temporal shifts of the maximum nutation frequency over the course of the measurements which show up as shifts of the peak of the nutation spectrum. In addition, sidebands of the inversion profiles spaced by the MAS frequency are visible.

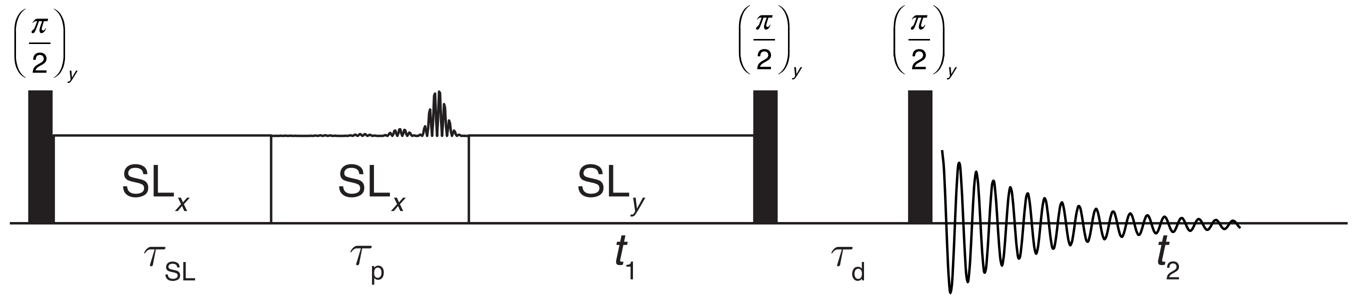

Figure S02: Schematic representation of the pulse sequence used for testing of the inversion properties of the I-BURP-2 pulse in the spin-lock frame with an additional spin-lock period before the inversion pulse. After the initial 90° pulse, the magnetization is spin locked along *x* for a time $\boldsymbol{\tau_{SL}}$ and the modulated I-BURP-2 inversion pulse is applied afterwards along *y*. During the subsequent t_1 time the magnetization nutates about the field along *y*. To obtain pure-phase spectra, a *z* filter with a dephasing delay is used to select a single component after the nutation. Difference spectra can be obtained by replacing the I-BURP-2 pulse in the spin-lock frame with a simple spinlock in alternating scans while shifting the receiver phase by 180°.

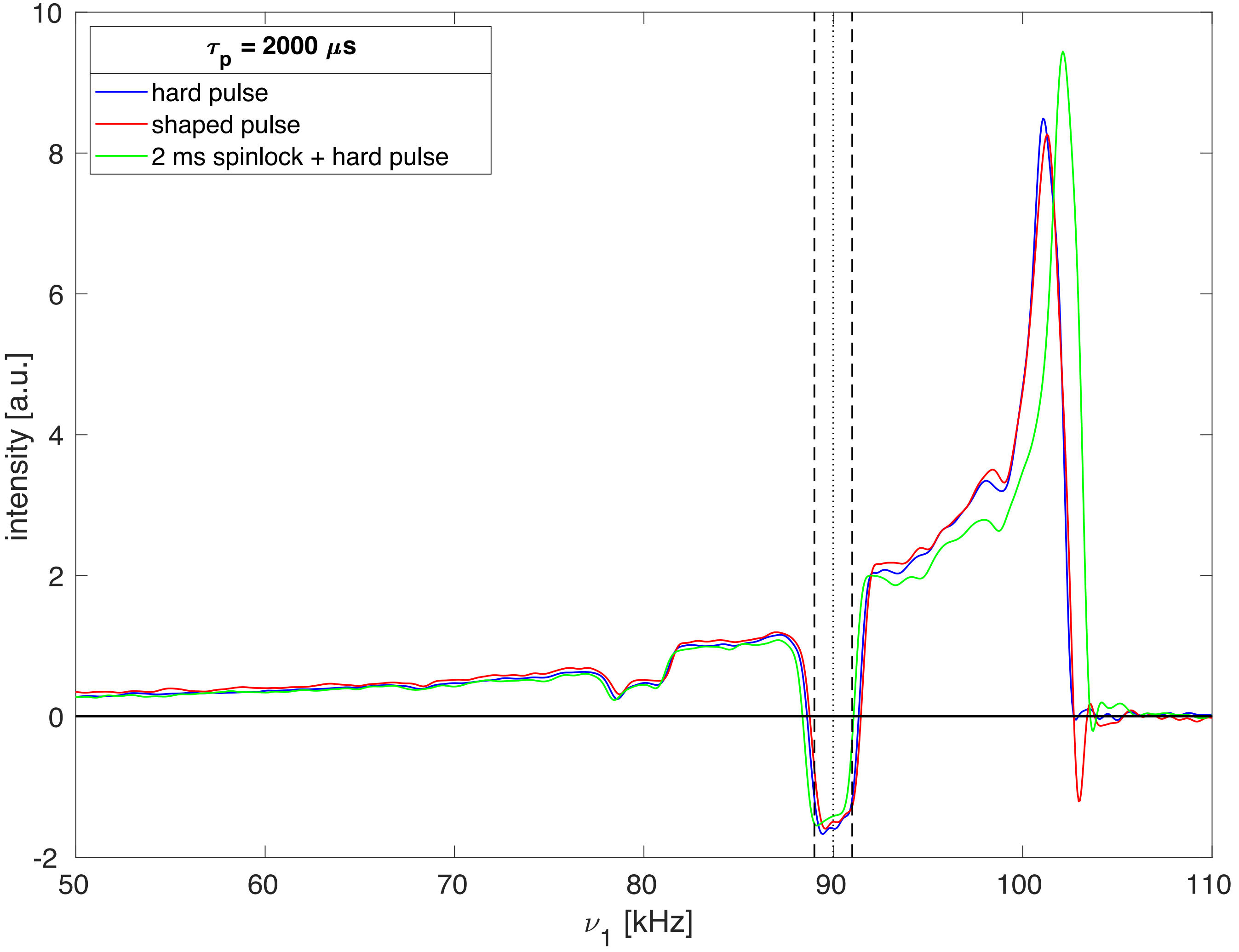

Figure S03: Proton nutation spectra of adamantane spinning at 10 kHz at 600 MHz proton resonance frequency using a Bruker 2.5 mm MAS probe. The nutation spectra were preceded by an I-BURP-2 pulse of length 2000 µs using a modulation frequency of 90 kHz. The nutation spectra were recorded using a hard pulse (blue and green spectra) while the nutation was implemented using a Bruker shaped pulse at 50% amplitude (red spectrum) with a scaling of the rf power by a factor of four. For the red spectrum, a spin-lock pulse of 2 ms was added before the I-BURP-2 pulse (see Fig. S02 for the pulse sequence). One can clearly see shifts of the maximum of the nutation spectrum depending on presence or absence of the additional spin-lock pulse. This is a known problem with current-generation Bruker power amplifiers and can explain the shift of the inverted area between theory and experiments as observed in Fig. 4 of the main paper.

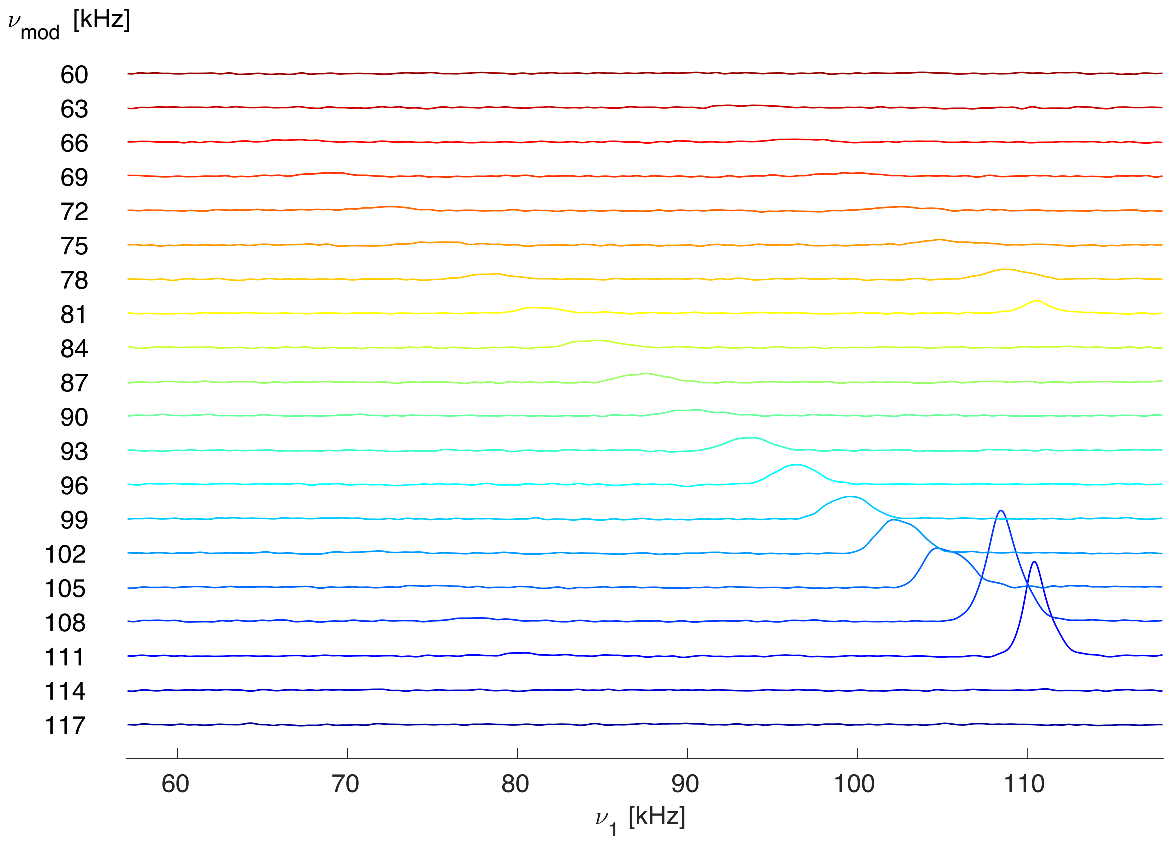

Figure S04: Separated slices of the nutation spectra of Fig. 5b) with their corresponding modulation frequencies.

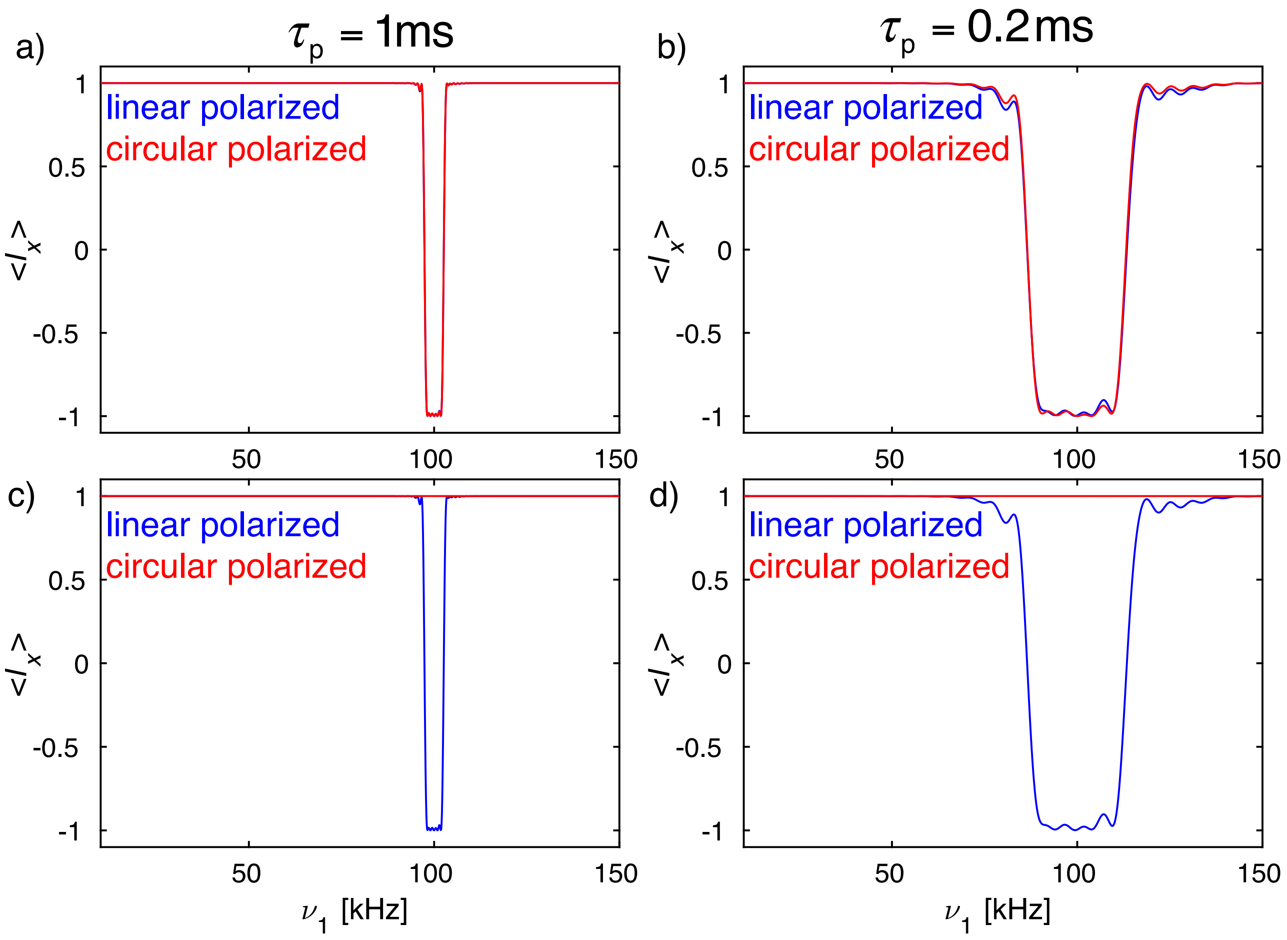

Figure S05: Simulation of the inversion efficiency as a function of the spin-lock amplitude. The blue line is the same simulation as the one shown in Figure 2 of the main paper using linear-polarized rf irradiation with a Hamiltonian given by $\mathcal{H}'(t) = \omega_1 I_x + 2\omega_2(t)\cos(\omega_{\text{mod}}t)\, I_y$ in the spin-lock frame. The red lines show the same simulations for circular polarized rf irradiation with a Hamiltonian given by $\mathcal{H}'(t) = \omega_1 I_x + \omega_2(t)\big(\cos(\omega_{\text{mod}}t)\, I_y \pm \sin(\omega_{\text{mod}}t)\, I_z\big)$, the top row with a positive sign, the bottom row with a negative sign. One can clearly see that only one of the two circular-polarized fields works and that there are only small differences between circular and linear polarized radio frequency.

Table S1: Experimental parameters of the homonuclear decoupling measurements shown in the paper.

**Proton Resonance Frequency [MHz]**	**Sample**	**Figure**	**Points: t_1**	**Spectral Width t_1 [kHz]**	**Points: t_2**	**Spectral Width t_2 [kHz]**	**No. of Scans**
500	Glycine	Fig. 7a)	512	12.5	1024	200	8
500	β-Asp-Ala	Fig. 7b)	768	12.5	1024	200	8
500	L-histidine·HCl·H_2O	Fig. 7c)	512	15.6	1024	200	8
600	L-histidine·HCl·H_2O (carrier at edge of spectrum)	Fig. 8	512	20.8	1024	200	8
600	L-histidine·HCl·H_2O (carrier in centre of spectrum)	Fig. 8	350	10.5	1024	200	16

Matlab Processing Scripts

2D Nutation Experiments

```
%proc_nutation_kaab.m
%K. Aebischer, 10.06.20

%Script to process raw data of 2D nutation experiment
%based on proc_nutation.m by M. Ernst

%Path to include proc_fid.m
addpath('../processing_scripts')

%Parameters
input_file ='./200622_His_nutation_600MHz/5/ser';

td2 = 1024;                    %time domain direct dimension
td1 = 512;                     %time domain indirect dimension
si2 = 4096;                    %zero-filling direct dimensuin
si1 = 4096;                    %zero-filling indirect dimension

swh1 = 1/(3.5e-6);             %Hz, spectral width indirect dimension
swh2 = 200000;                 %Hz, spectral width direct dimension

%Phase correction
p0_2 = -58;                   %zero order direct dimension
p1_2 = -3;                    %first order direct dimension
p0_1 = 90;                    %zero order indirect dimension
p1_1 = 90;                    %first order indirect dimension

%Read data from input file
fid = fopen(input_file,'r','l');
a = fread(fid,2*td1*td2,'int32');
fclose(fid);

%Reshape into 2D array
a1 = reshape(a,2*td2,td1);
a2 = zeros(td2,td1);
%Combine to complex number
for k=1:td1
  a2(:,k) = a1(1:2:end,k) + 1i*a1(2:2:end,k);
end

%Process FIDs in direct dimension
a2p=zeros(si2,td1);
for k = 1:td1
    [a2p(:,k), ~] = proc_fid(a2(1:end,k), swh2, si2, 0, p0_2, p1_2, 2, 2, si2/2, 76);
end

%FT along indirect dimension
%discard imaginary part
%sum over relevant part in omega_2
[spectrum, frq_ax] = proc_fid(sum(real(a2p(900:3000, :))), swh1, si1, 0, p0_1, p1_1, 2, 2);
```

```
%proc_pmlg_kaab.m
%K. Aebischer 19.06.20

%Script to process raw data of 2D homonuclear decoupled proton spectra

%Path to include prod_fid.m
addpath('../processing_scripts')

%Parameters
input_file = './200626_AlaAsp_PMLG/20/ser';
td2 = 1024;          %time domain direct dimension
td1 = 512;           %time domain indirect dimension
si2 = 4096;          %zero-filling direct dimension
si1 = 4096;          %zero-filling indirect dimensin
swh1 = 12.5e3;       %spectral width indirect dimension, Hz
swh2 = 200e3;        %spectral width direct dimension, Hz

%Phase correction
p0_2 = 110;                  %zero order direct dimension
p1_2 = 3;                   %first order direct dimension
p0_1 = 90;                   %zero order indirect dimension
p1_1 = 0;                   %first order indirect dimension

%Read data from input file
fid = fopen(input_file,'r','l');
a = fread(fid,2*td1*td2,'int32');
fclose(fid);

%Reshape into 2D array
a1=reshape(a,2*td2,td1);
a2 = zeros(td2,td1);
%Combine to complex number
for k=1:td1
    a2(:,k) = a1(1:2:end,k)+1i*a1(2:2:end,k);
end

%Process FIDs in direct dimension
a2p=zeros(si2,td1);
for k=1:td1
    [a2p(:,k), ~] = proc_fid(a2(:,k),swh2,si2,0,p0_2,p1_2,0,2,2048,76);
end

%Sum of relevant part in omega_2
a1fr = sum(real(a2p(600:3400, :)));
a1fc = a1fr(1:2:td1)+1i*a1fr(2:2:td1);

%FT along second dimension
[spectrum, frq_ax] = proc_fid(a1fc,swh1,si1,0,p0_1,p1_1,2,2);
```

FID Processing

```
function [data_ft, frq_ax] = proc_fid(data,sw,zf,lb,phase0, phase1, basl, win, zp, fs)
%K. Aebischer, 10.06.20
%based on phase1.m by M. Ernst
%Function for basic processing of FID signal including zero-filling,
%baseline and phase correction and apodization
%[data_ft, frq_ax] = proc_fid(data,sw,zf,lb,phase0, phase1, basl, win, zp, fs)
%
%Input
    %data:      array with FID datapoints
    %sw:        spectral width in Hz
    %zf:        Zero-filling
    %lb:        line-broadening (Hz) for exponential multiplication
    %phase0:    deg, 0 order phase correction
    %phase1:    deg, 1st order phase correction
    %basl:      option for baseline correction (0: none, 1: on FID, 2: FID and spectrum)
    %win:       apodization window (0: exponential, else: cos^2)
    %zp:        zero-point for first order phase correction (point index)
    %fs:        Shift FID by fs points (protection delay)
%Output:
    %data_ft:   spectrum of FID
    %frq_ax:    frequency axis of spectrum in Hz

data=data(:).';         %ensures data is a row vector
dw = 1/sw;              %dwell time (time-res. of FID)
l=length(data);

%Check input arguments
%set default values if no argument given
if nargin < 3
    zf=1;
end
if nargin < 4
    lb=0;
end
if nargin < 5
    phase0=0;
end
if nargin < 6
    phase1=0;
end
if nargin < 7
    basl=0;
end
if nargin < 8
    win=0;
end
if nargin < 9
    zp=zf/2;
end
if nargin < 10
    fs=0;
end
```

```
phase1 = phase1-fs*180;
if zf==0
  zf = l;
end

%Offset correction FID
%takes the last 20% of the FID and corrects by mean value
if basl > 0
  offset = mean(data(round(0.8*l):l));
  data(fs+1:l) = data(fs+1:l) - offset;
end

%Apodization
if win == 0
%exponential window
    apod = ones(1,l);
    apod(fs+1:l) = exp(-lb*(0:(l-(fs+1)))*dw*pi);
else
%cos^2 window
    apod = ones(1,l);
    apod(fs+1:l) = cos((0:(l-(fs+1)))/(l-(fs+1))*pi/2).^2;
end
data = data .* apod;
data(1)=0.5*data(1);

%Fourier tranform FID
data_ft = fftshift(fft(data,zf));

%0 order phase correction
data_ft = data_ft * exp(-1i*pi/180*phase0);

%1st order phase correction
x = (((0:(zf-1))-zp+zf/2)/zf)-0.5;
frq_ax = x*sw;
data_ft = data_ft .* exp(-1i*pi/180*2*x*phase1);

%Offset correction for spectrum
if basl > 1
%use edge of spectral width for baseline correction
    offset = mean(data_ft(round(0.1*l):round(0.2*l)));
    data_ft = data_ft - offset;
end

end
%end of function
```
